# Epigenetic changes within the promoter region of the *HLA-G *gene in ovarian tumors

**DOI:** 10.1186/1476-4598-7-43

**Published:** 2008-05-22

**Authors:** Laura Menendez, L DeEtte Walker, Lilya V Matyunina, Kimberly A Totten, Benedict B Benigno, John F McDonald

**Affiliations:** 1 Department of Genetics, University of Georgia, Athens, GA 30605, USA; 2 School of Biology, Georgia Institute of Technology, Atlanta, GA 30332, USA; 3 Ovarian Cancer Institute, Atlanta, GA 30342, USA

## Abstract

**Background:**

Previous findings have suggested that epigenetic-mediated *HLA-G *expression in tumor cells may be associated with resistance to host immunosurveillance. To explore the potential role of DNA methylation on *HLA-G *expression in ovarian cancer, we correlated differences in *HLA-G *expression with methylation changes within the *HLA-G *regulatory region in an ovarian cancer cell line treated with 5-aza-deoxycytidine (5-aza-dC) and in malignant and benign ovarian tumor samples and ovarian surface epithelial cells (OSE) isolated from patients with normal ovaries.

**Results:**

A region containing an intact hypoxia response element (HRE) remained completely methylated in the cell line after treatment with 5-aza-dC and was completely methylated in all of the ovarian tumor (malignant and benign) samples examined, but only variably methylated in normal OSE samples. *HLA-G *expression was significantly increased in the 5-aza-dC treated cell line but no significant difference was detected between the tumor and OSE samples examined.

**Conclusion:**

Since HRE is the binding site of a known repressor of *HLA-G *expression (HIF-1), we hypothesize that methylation of the region surrounding the HRE may help maintain the potential for expression of *HLA-G *in ovarian tumors. The fact that no correlation exists between methylation and *HLA-G *gene expression between ovarian tumor samples and OSE, suggests that changes in methylation may be necessary but not sufficient for *HLA-G *expression in ovarian cancer.

## Background

Classic and non-classic HLA (human leukocyte antigen) class I genes play a central role in the regulation of the immune response. The non-classic *HLA-G *gene is expressed in a variety of tissues but perhaps most notably in the fetal-maternal interface on the extravillous cytotrophoblast and has been postulated to help protect the fetus from maternal allorecognition [1]. This hypothesis is supported by subsequent studies demonstrating that *HLA-G *proteins can suppress a variety of immune functions including natural killer (NK) cell-mediated cytolysis and the T-cell proliferative response [2,3]. Recent findings indicate that *HLA-G *antigens are present in ovarian and various other types of malignant cells and tissues [4-7]. These findings and others have led to the hypothesis that induction of *HLA-G *expression in tumor cells may contribute to their avoidance of immunosurveillance by the host [8,9] (but disputed by [10]).

Sequences known to be involved in the transcriptional regulation of most HLA class I genes are disrupted in the *HLA-G *gene raising questions as to the mechanisms underlying *HLA-G *expression [11-13]. Studies conducted in a variety of human cancer cell lines suggest that epigenetic mechanisms may play an important role in *HLA-G *expression [14,15]. To explore the potential role of DNA methylation on *HLA-G *expression in ovarian cancer, we tested the effect of the methylation inhibitor 5-aza-deoxycytidine on methylation within the CpG-enriched regulatory region of the *HLA-G *gene and correlated changes in expression in an ovarian cancer cell line. The results demonstrate that 5-aza-dC treatment results in hypomethylation of putative control sequences within the 5' regulatory region of *HLA-G *and that these changes in methylation correlate with a significant increase in expression. A notable exception was a region (-211 to -290) containing a hypoxia response element (HRE; [16]) that remained completely methylated.

Methylation within the regulatory region of the *HLA-G *gene also was examined in eighteen malignant and benign ovarian tumor samples and in ovarian surface epithelial cells (OSE) isolated from four patients with normal ovaries. A number of significant differences in levels of methylation of sequences within the 5' regulatory region were detected between the tumor samples and the normal surface epithelial cells. Interestingly, the region containing the HRE (-211 to -290) that remained methylated in 5-aza-dC treated BG-1 cells was also completely methylated in all ovarian tumor samples, but not in OSE controls, suggesting strong selection against accessibility to the HRE in ovarian tumor cells. Although the highest levels of *HLA-G *expression were associated with tumor samples, no significant overall correlation between methylation and expression levels was detected by real time RT-PCR. Our results indicate that alterations in methylation may be necessary but not sufficient for *HLA-G *expression in ovarian tumors.

## Results

### 5-aza-dC treatment of ovarian cancer cells (BG-1) results in hypomethylation of sequences within the *HLA-G *regulatory region and correlates with an increase in gene expression

**Figure 1:**
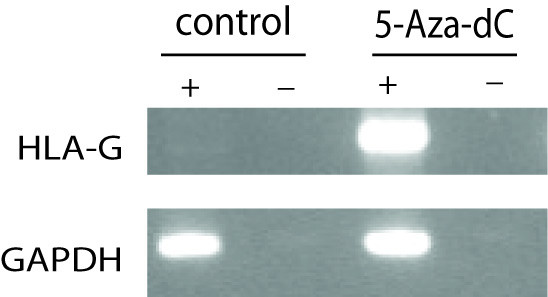
**Activation of *HLA-G *gene transcription in the BG-1 cell line treated with 5-aza-deoxycytidine (5-aza-dC)**. Results of semi-quantitative RT-PCR (reverese transcriptase-polymerase chain reaction) on the ovarian carcinoma cell line BG-1, either untreated (control) or treated with 50 μM 5-aza-deoxycytidine (5-aza-dC). ("-" = samples without RT; "+" = samples with RT). *GAPDH *was used as an endogenous control.

**Figure 2:**
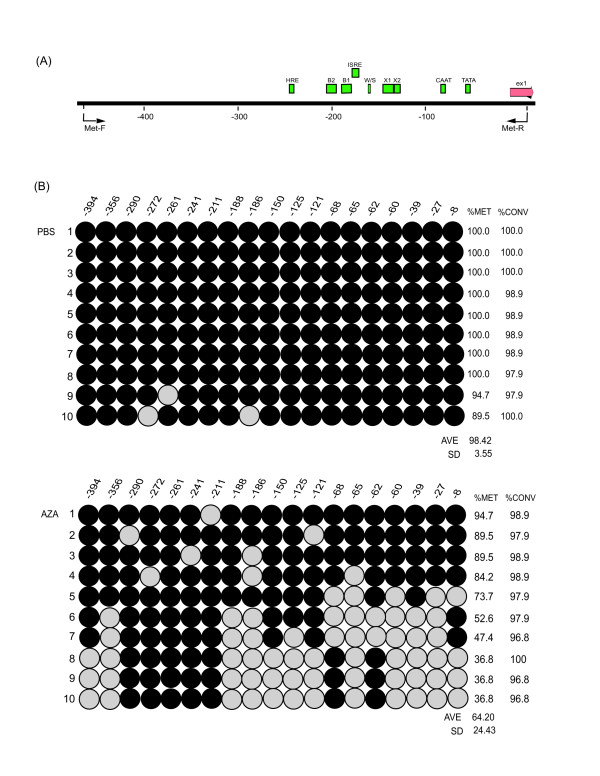
**Methylation analysis of the *HLA-G *promoter in the control and 5-aza-dC treated BG-1 cell line**. **(A) **Schematic map of 450 bp of the *HLA-G *promoter region. Colored boxes represent enhancers and regulator binding sites: HRE = hypoxia response element; B2 = enhancer κB2; B1 = enhancer κB1; ISRE = interferon sequence responsive element; W/S= W/S box; X1 = conserved X1 regulatory box; X2 = X2 box; CAAT = CCAAT box; TATA = TATA box; ex1 = exon 1; Met-F and Met-R = forward (F) and reverse (R) primer binding sites. **(B) **Bisulfite genomic sequencing of 19 CpG dinucleotides of the region from -450 to ATG. Individual CpG dinulcotides are depicted as circles. Each row of circles represents an individual sequenced clone, either untreated (PBS) or treated with 50 μM 5-aza-dC (open circle = 100% unmethylated; filled circle = 100% methylated; %MET = percentage of methylation of each individual clone; %CONV = efficiency of sodium bisulfite treatment).

Previous studies have shown that 5-aza-deoxycytidine treatment of a variety of tumor (glioma, choriocarcinoma, B-lymphoma and melanoma) cell lines results in significant hypomethylation of a CpG-rich region located within 450 bp 5' of the *HLA-G *start codon and correlates with a significant increase in *HLA-G *expression [15]. To determine if ovarian tumor cells would show a similar response, we selected an ovarian cancer cell line (BG-1) that did not display *HLA-G *expression prior to treatment (Figure 1). Sodium bisulfite modification and subsequent sequencing was carried out on 10 clones each of genomic DNA isolated from 5-aza-dC treated and untreated BG-1 cells. The results demonstrate that while all 19 CpG sites located within the 450 bp region 5' of the *HLA-G *start codon were methylated in untreated cells, there was a 36% overall decrease of methylation in treated cells (Figure 2). The approximately 200 bp region immediately 5' to the transcriptional start site (-8 to -188) that encompasses *HLA-G cis*-regulatory sequences was significantly (p = 0.001) hypomethylated, but a 79 bp region (-211 to -290) remained methylated after 5-aza-dC treatment in all of the clones examined. Correlated with the 5-aza-dC induced changes in methylation patterns, there was an increase in *HLA-G *expression (Figure 1).

### Methylation within the *HLA-G *5' regulatory region significantly differs between ovarian surface epithelial cells (OSEs) and benign and malignant ovarian tumors

**Figure 3:**
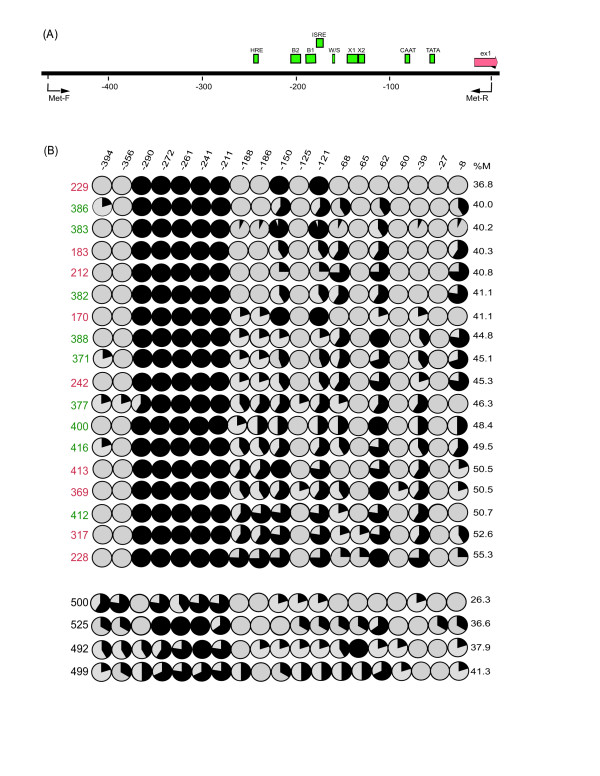
**Methylation analysis of the *HLA-G *promoter in tumor and normal ovarian samples**. **(A) **Schematic map of 450 bp of the *HLA-G *promoter region (See Figure 2 legend for symbols). **(B**) Bisulfite genomic sequencing of 19 CpG dinucleotides of the region from -450 to the transcription start site is analyzed for 18 ovarian adenocarcinomas (red) and benign adenomas (green) and four normal ovaries (black). Five to eight clones were sequenced for each sample and each circle represents the average methylation for a single CpG dinucleotide (open circle = 100% unmethylated; filled circle = 100% methylated; %M = percentage of total methylation per sample).

Epithelial ovarian carcinomas are believed to derive from ovarian surface epithelial cells (OSE) or epithelial cells lining the inner surface of inclusion cysts [17,18]. To determine if methylation within the 5' regulatory region of the *HLA-G *gene differs among OSE and benign and malignant ovarian tumors, we performed sodium bisulfite genomic sequencing of DNA isolated from laser capture microdissected (LCM) tumor cells from 9 adenomas and 9 adenocarcinomas and OSE brushings from the normal ovaries of 4 patients undergoing hysterectomies for reasons unrelated to ovarian cancer. Five to eight clones were sequenced from each DNA sample. The average % methylation for all 19 CpG sites located from 8 to 394 bp 5' to the *HLA-G *transcriptional start site is presented in Table 1 and Figure 3.

**Table 1:** Methylation status of 19 CpG sites in the 450 bp promoter region of *HLA-G *gene in malignant (CA), benign (BN) and normal (NL) ovarian tissue samples.

CA	-394	-356	-290	-272	-261	-241	-211	-188	-186	-150	-125	-121	-68	-65	-62	-60	-39	-27	-8
212	0	0	1	1	1	1	1	0.00	0.00	0.25	0.00	0.25	0.75	0.00	0.75	0.00	0.00	0.00	0.75
229	0	0	1	1	1	1	1	0.00	0.00	1.00	0.00	1.00	0.00	0.00	0.00	0.00	0.00	0.00	0.00
183	0	0	1	1	1	1	1	0.00	0.00	0.38	0.00	0.38	0.63	0.00	0.63	0.00	0.00	0.00	0.63
317	0	0	1	1	1	1	1	0.60	0.60	0.80	0.00	0.80	0.20	0.20	0.80	0.00	0.60	0.00	0.40
369	0	0	1	1	1	1	1	0.40	0.40	0.60	0.20	0.60	0.40	0.00	1.00	0.20	0.60	0.00	0.20
413	0	0	1	1	1	1	1	0.60	0.60	1.00	0.00	0.80	0.00	0.00	0.80	0.00	0.60	0.00	0.20
170	0	0	1	1	1	1	1	0.20	0.20	1.00	0.00	1.00	0.00	0.00	0.20	0.00	0.20	0.00	0.00
228	0	0	1	1	1	1	1	0.75	0.75	0.75	0.00	0.75	0.25	0.25	1.00	0.00	0.75	0.00	0.25
242	0	0	1	1	1	1	1	0.20	0.20	0.40	0.00	0.40	0.60	0.00	0.80	0.00	0.20	0.00	0.80

**AVE**	**0.000**	**0.000**	**1.000**	**1.000**	**1.000**	**1.000**	**1.000**	**0.306**	**0.306**	**0.687**	**0.022**	**0.664**	**0.314**	**0.050**	**0.664**	**0.022**	**0.328**	**0.000**	**0.359**

BN																			

382	0	0	1	1	1	1	1	0.00	0.00	0.40	0.00	0.40	0.60	0.00	0.60	0.00	0.00	0.00	0.80
412	0	0	1	1	1	1	1	0.60	0.80	0.80	0.00	0.80	0.20	0.00	0.83	0.00	0.60	0.00	0.00
388	0	0	1	1	1	1	1	0.17	0.17	0.17	0.00	0.17	0.67	0.00	1.00	0.00	0.33	0.00	0.83
416	0.20	0	1	1	1	1	1	0.40	0.40	0.60	0.00	0.60	0.40	0.00	0.80	0.00	0.40	0.00	0.60
371	0.14	0	1	1	1	1	1	0.14	0.14	0.43	0.00	0.43	0.57	0.00	0.71	0.00	0.29	0.00	0.71
377	0.20	0.20	0.60	1	1	1	1	0.40	0.60	0.60	0.20	0.60	0.20	0.00	0.60	0.00	0.60	0.00	0.00
386	0.20	0	1	1	1	1	1	0.00	0.00	0.60	0.00	0.60	0.40	0.00	0.40	0.00	0.00	0.00	0.40
400	0	0	1	1	1	1	1	0.20	0.50	0.50	0.00	0.50	0.50	0.00	1.00	0.00	0.50	0.00	0.50
383	0	0	1	1	1	1	1	0.10	0.10	0.90	0.00	0.90	0.10	0.00	0.30	0.00	0.10	0.00	0.13

**AVE**	**0.082**	**0.022**	**0.956**	**1.000**	**1.000**	**1.000**	**1.000**	**0.223**	**0.301**	**0.556**	**0.022**	**0.556**	**0.404**	**0.000**	**0.694**	**0.000**	**0.313**	**0.000**	**0.441**

NL																			

492	0.40	0.40	0.40	0.60	0.80	1.00	0.80	0.00	0.20	0.20	0.20	0.20	0.40	1.00	0.20	0.20	0.00	0.00	0.20
499	0.17	0.33	0.50	0.67	0.83	0.67	0.83	0.50	0.00	0.33	0.50	0.50	0.50	0.50	0.67	0.17	0.00	0.00	0.17
500	0.60	0.80	0.00	0.80	0.40	0.80	0.80	0.00	0.00	0.20	0.20	0.20	0.00	0.00	0.00	0.00	0.20	0.00	0.00
525	0.33	0.33	0.00	1.00	1.00	1.00	0.67	0.00	0.00	0.00	0.33	0.33	0.33	0.33	0.67	0.00	0.00	0.33	0.33
**AVE**	**0.374**	**0.466**	**0.225**	**0.767**	**0.758**	**0.867**	**0.776**	**0.125**	**0.050**	**0.183**	**0.308**	**0.308**	**0.308**	**0.458**	**0.384**	**0.092**	**0.050**	**0.083**	**0.174**

P-VALUES																			

CA VS NL	<1E-04	<1E-04	<1E-04	0.001	0.011	0.025	<1E-04	0.308	0.122	0.008	0.0004	0.033	0.970	0.014	0.225	0.174	0.111	0.139	0.277
BN VS NL	0.003	0.0002	<1E-04	0.001	0.011	0.025	<1E-04	0.466	0.121	0.010	0.0004	0.063	0.448	0.005	0.087	0.019	0.059	0.139	0.153

Values shown for each sample/position are the average of 5–8 clones. Significance of differences at each CpG site between tumor and normal samples are shown.

Average methylation levels across the 450 bp 5' region examined were variable among samples, ranging from 26.3% to 55.3%. The malignant and benign tumor samples displayed nearly identical average methylation levels across the region (adenocarcinomas 45.9%; adenomas 45.1%) while average methylation levels (35.5%) were significantly lower for the OSE samples (adenocarcinoma vs. OSE, p = 0.022; adenoma vs. OSE, p = 0.007, unpaired t-test). The same region (CpG sites -211 to -290) that remained methylated after 5-aza-dC treatment in the BG-1 cell line was uniformly hypermethylated in all of the tumor samples, but variably methylated in the OSE samples.

Examination of the methylation levels between the benign and malignant tumor samples at each of the 19 individual CpG sites displayed no significant differences (Table 1). However, 12 of the 19 sites displayed significant differences between the OSE and tumor samples (Table 1). The OSE samples displayed significantly higher levels of methylation than tumor samples at 5 (-60, -65, -125, -356 and -394) of the 19 CpG sites examined. OSE samples displayed significantly lower levels of methylation than tumor samples at 7 (-121, -150, -211, -241, -261, -272, and -290) of the 19 CpG sites examined.

We also examined a second 399 bp region of the *HLA-G *CpG island from -7 to + 392 extending into Exon 2. This region contains 42 CpG dinucleotides. No significant difference overall in levels of methylation between OSE and malignant or benign ovarian tumor samples was detected within this region (p < 0.05; Table 2).

**Table 2:** Methylation status of 42 CpG sites in a 399 bp region of *HLA-G *gene extending from the transcriptional start site (TSS) into Exon II (-7 to +392) in malignant (CA), benign (BN) and normal (NL) ovarian tissue samples.

CA	+18	+40	+59	+70	+80	+97	+106	+108	+124	+129	+132	+138	+147	+154	+156	+159	+171
212	0.63	0.50	0.50	0.50	0.50	0.75	0.63	0.63	0.50	0.00	0.50	0.50	0.50	0.50	0.50	0.50	0.50
183	0.50	0.50	0.50	0.50	0.50	0.00	0.50	0.50	0.50	0.00	0.50	0.50	0.50	0.50	0.50	0.50	0.50
369	0.80	0.90	0.90	0.80	0.80	0.80	0.80	0.80	0.58	0.00	0.80	0.70	0.68	0.80	0.68	0.80	0.68
242	0.38	0.39	0.45	0.39	0.32	0.25	0.13	0.32	0.32	0.25	0.32	0.32	0.32	0.32	0.32	0.32	0.32
170	1.00	0.60	1.00	1.00	1.00	1.00	1.00	1.00	0.88	0.00	1.00	1.00	1.00	1.00	1.00	1.00	1.00
413	1.00	1.00	1.00	1.00	0.93	0.50	1.00	0.88	1.00	0.00	1.00	1.00	1.00	0.88	1.00	1.00	1.00
317	0.88	0.88	0.88	0.88	0.88	0.50	0.88	0.88	0.88	0.00	0.88	0.88	0.88	0.88	0.88	0.88	0.88
228	1.00	1.00	1.00	1.00	1.00	1.00	1.00	1.00	1.00	0.00	0.50	1.00	1.00	1.00	1.00	1.00	1.00

**AVE**	**0.77**	**0.72**	**0.78**	**0.76**	**0.74**	**0.60**	**0.74**	**0.75**	**0.71**	**0.03**	**0.69**	**0.74**	**0.73**	**0.73**	**0.73**	**0.75**	**0.73**

BN																	

382	0.58	0.71	0.58	0.58	0.46	0.50	0.58	0.58	0.73	0.00	0.58	0.58	0.58	0.58	0.58	0.58	0.58
412	1.00	1.00	1.00	1.00	1.00	0.75	1.00	1.00	0.75	0.13	0.63	0.75	1.00	1.00	1.00	1.00	1.00
388	1.00	1.00	1.00	1.00	1.00	0.50	1.00	0.63	1.00	0.00	1.00	1.00	1.00	1.00	1.00	1.00	1.00
416	0.50	0.67	0.50	0.50	0.50	0.50	0.50	0.50	0.50	0.00	0.50	0.50	0.17	0.50	0.50	0.50	0.50
371	1.00	1.00	1.00	1.00	1.00	1.00	1.00	1.00	0.83	0.17	1.00	1.00	1.00	1.00	1.00	1.00	1.00
377	1.00	1.00	1.00	1.00	1.00	0.38	1.00	0.88	0.75	0.17	0.88	0.88	0.88	1.00	1.00	0.88	1.00
386	1.00	1.00	1.00	1.00	0.88	0.88	1.00	1.00	0.71	0.50	0.83	0.83	0.83	1.00	1.00	1.00	1.00
400	1.00	1.00	1.00	1.00	1.00	1.00	0.67	0.67	0.70	0.00	0.50	1.00	1.00	1.00	1.00	1.00	1.00
383	1.00	1.00	1.00	1.00	1.00	0.00	1.00	1.00	1.00	0.00	0.00	1.00	1.00	1.00	1.00	1.00	1.00

**AVE**	**0.90**	**0.93**	**0.90**	**0.90**	**0.87**	**0.61**	**0.86**	**0.81**	**0.78**	**0.11**	**0.66**	**0.84**	**0.83**	**0.90**	**0.90**	**0.88**	**0.90**

NL																	

500	1.00	0.83	0.83	0.83	1.00	0.67	0.83	0.83	1.00	0.00	0.83	0.83	0.50	0.50	0.33	0.50	0.50
525	0.75	0.75	0.63	0.75	0.75	0.50	0.75	0.88	0.75	0.00	0.75	0.75	0.75	0.75	0.75	0.75	0.63
492	0.63	0.50	0.50	0.50	0.50	0.25	0.63	0.63	0.63	0.00	0.63	0.50	0.63	0.63	0.63	0.63	0.75
499	0.75	0.71	0.58	0.71	0.58	0.29	0.88	0.88	0.54	0.00	0.71	0.71	0.88	0.58	0.71	0.71	0.88

AVE	**0.78**	**0.70**	**0.64**	**0.70**	**0.71**	**0.43**	**0.77**	**0.80**	**0.73**	**0.00**	**0.73**	**0.70**	**0.69**	**0.61**	**0.60**	**0.65**	**0.69**

P-VALUES																	

CA VS NL	0.946	0.869	0.322	0.673	0.838	0.390	0.857	0.690	0.879	0.506	0.766	0.795	0.762	0.403	0.415	0.487	0.762
BN VS NL	0.332	0.018	0.041	0.105	0.255	0.320	0.446	0.990	0.660	0.235	0.671	0.220	0.380	0.025	0.032	0.051	0.10
CA	+175	+193	+227	+230	+233	+240	+245	+249	+251	+261	+269	+281	+287	+292	+299	+303	+308

212	0.50	0.50	0.50	0.50	0.50	0.50	0.50	0.38	0.00	0.50	0.50	0.50	0.50	0.50	0.38	0.50	0.50
183	0.50	0.50	0.50	0.50	0.50	0.50	0.50	0.50	0.50	0.50	0.50	0.50	0.50	0.50	0.50	0.50	0.50
369	0.58	0.68	0.80	0.80	0.80	0.80	0.80	0.90	0.80	0.70	0.80	0.80	0.80	0.80	0.80	0.80	0.68
242	0.32	0.32	0.46	0.39	0.39	0.39	0.32	0.32	0.32	0.39	0.46	0.32	0.32	0.32	0.20	0.32	0.39
170	1.00	1.00	1.00	1.00	1.00	1.00	1.00	1.00	1.00	0.63	1.00	1.00	1.00	1.00	1.00	1.00	1.00
413	1.00	1.00	0.88	0.88	0.88	0.88	0.88	0.75	0.88	0.75	0.88	0.88	0.63	1.00	1.00	1.00	1.00
317	0.88	0.38	1.00	1.00	1.00	1.00	1.00	1.00	1.00	1.00	1.00	1.00	1.00	0.50	1.00	1.00	1.00
228	1.00	1.00	1.00	1.00	1.00	1.00	1.00	1.00	1.00	1.00	1.00	1.00	1.00	1.00	1.00	1.00	1.00

**AVE**	**0.72**	**0.67**	**0.77**	**0.76**	**0.76**	**0.76**	**0.75**	**0.73**	**0.69**	**0.68**	**0.77**	**0.75**	**0.72**	**0.70**	**0.73**	**0.77**	**0.76**

BN																	

382	0.58	0.58	0.58	0.58	0.58	0.58	0.58	0.58	0.46	0.58	0.71	0.58	0.46	0.58	0.58	0.58	0.58
412	0.63	1.00	1.00	1.00	1.00	1.00	1.00	1.00	1.00	1.00	1.00	1.00	1.00	1.00	1.00	1.00	1.00
388	1.00	1.00	1.00	1.00	1.00	1.00	1.00	1.00	1.00	0.75	1.00	1.00	1.00	1.00	1.00	1.00	1.00
416	0.50	0.50	0.50	0.50	0.50	0.50	0.50	0.50	0.50	0.50	0.50	0.50	0.50	0.50	0.50	0.50	0.67
371	1.00	1.00	1.00	1.00	1.00	1.00	1.00	1.00	1.00	0.17	1.00	1.00	1.00	1.00	1.00	1.00	1.00
377	1.00	0.88	1.00	1.00	1.00	1.00	1.00	0.88	1.00	0.88	1.00	1.00	0.88	1.00	1.00	1.00	1.00
386	1.00	1.00	1.00	1.00	1.00	1.00	1.00	1.00	1.00	0.83	1.00	1.00	1.00	1.00	1.00	1.00	0.88
400	1.00	1.00	1.00	1.00	1.00	0.70	1.00	1.00	1.00	1.00	1.00	1.00	1.00	1.00	1.00	1.00	1.00
383	1.00	1.00	1.00	1.00	1.00	1.00	1.00	1.00	1.00	1.00	1.00	1.00	1.00	1.00	1.00	1.00	1.00

**AVE**	**0.86**	**0.88**	**0.90**	**0.90**	**0.90**	**0.86**	**0.90**	**0.88**	**0.88**	**0.75**	**0.91**	**0.90**	**0.87**	**0.90**	**0.90**	**0.90**	**0.90**

NL																	

500	0.50	0.50	0.50	0.50	0.50	0.50	0.50	0.50	0.50	0.50	0.50	0.50	0.83	0.50	0.50	0.50	0.50
525	0.75	0.63	0.75	0.75	0.75	0.75	0.75	0.75	0.75	0.75	0.75	0.88	0.63	0.63	0.75	0.75	0.75
492	0.75	0.88	0.75	0.75	0.63	0.75	0.75	0.75	0.75	0.75	0.75	0.75	0.75	0.75	0.63	0.75	0.63
499	0.88	0.88	0.88	0.88	0.88	0.88	0.88	0.88	0.88	0.88	0.88	0.88	0.88	0.88	0.88	0.88	0.88

AVE	**0.72**	**0.72**	**0.72**	**0.72**	**0.69**	**0.72**	**0.72**	**0.72**	**0.72**	**0.72**	**0.72**	**0.75**	**0.77**	**0.69**	**0.69**	**0.72**	**0.69**

P-VALUES																	

CA VS NL	0.986	0.776	0.725	0.784	0.640	0.784	0.840	0.941	0.877	0.788	0.725	0.998	0.720	0.923	0.799	0.780	0.650
BN VS NL	0.283	0.200	0.148	0.148	0.096	0.241	0.148	0.173	0.222	0.865	0.096	0.250	0.427	0.100	0.100	0.148	0.053
CA	+314	+319	+322	+328	+340	+342	+346	+349									

212	0.50	0.50	0.50	0.50	0.50	0.50	0.50	0.50									
183	0.50	0.50	0.50	0.50	0.50	0.50	0.50	0.50									
369	0.80	0.80	0.80	0.80	0.80	0.80	0.80	0.80									
242	0.32	0.39	0.32	0.45	0.45	0.45	0.45	0.45									
170	1.00	1.00	1.00	1.00	1.00	1.00	1.00	1.00									
413	1.00	1.00	1.00	1.00	1.00	1.00	1.00	1.00									
317	1.00	1.00	1.00	1.00	1.00	1.00	1.00	1.00									
228	1.00	1.00	1.00	1.00	1.00	1.00	1.00	1.00									

**AVE**	**0.77**	**0.77**	**0.77**	**0.78**	**0.78**	**0.78**	**0.78**	**0.78**									

BN																	

382	0.58	0.58	0.58	0.58	0.58	0.58	0.58	0.58									
412	1.00	1.00	1.00	1.00	1.00	1.00	1.00	1.00									
388	1.00	1.00	1.00	1.00	1.00	1.00	1.00	1.00									
416	0.50	0.50	0.50	0.50	0.50	0.50	0.50	0.50									
371	1.00	1.00	1.00	1.00	1.00	1.00	1.00	1.00									
377	1.00	1.00	1.00	1.00	1.00	1.00	1.00	1.00									
386	1.00	1.00	1.00	1.00	1.00	1.00	1.00	1.00									
400	1.00	1.00	0.83	1.00	1.00	1.00	1.00	1.00									
383	1.00	1.00	1.00	1.00	1.00	1.00	1.00	1.00									

**AVE**	**0.90**	**0.90**	**0.88**	**0.90**	**0.90**	**0.90**	**0.90**	**0.90**									

NL																	

500	0.50	0.50	0.50	0.33	0.50	0.50	0.50	0.50									
525	0.75	0.75	0.75	0.75	0.75	0.75	0.75	0.75									
492	0.75	0.75	0.75	0.75	0.75	0.75	0.75	1.00									
499	0.88	0.88	0.88	0.88	0.88	0.88	0.88	0.88									

AVE	**0.72**	**0.72**	**0.72**	**0.68**	**0.72**	**0.72**	**0.72**	**0.78**									

P-VALUES																	

CA VS NL	0.769	0.714	0.769	0.515	0.671	0.671	0.671	0.998									
BN VS NL	0.148	0.148	0.190	0.120	0.148	0.148	0.148	0.365									

Values shown for each sample/position are the average of 5–8 clones. Marginally significant differences between tumor and normal samples were detected at 4 of the 42 CpG sites.

### No significant difference in *HLA-G *expression was detected between OSE and malignant and benign ovarian tumor samples

To determine if levels of *HLA-G *expression are significantly different between ovarian tumors and OSE cells, we determined relative expression levels (real time RT-PCR) of 3 OSE samples and tumor cells isolated by LCM from 4 malignant and 4 benign ovarian tumor samples. The results presented in Table 3 indicate that *HLA-G *expression levels are highly variable among all samples and no consistent or significant differences in *HLA-G *expression were detected between the OSE and tumor samples. Our results are consistent with previous reports of high variability in *HLA-G *expression within and between tumors [7,10]. Thus, the significant difference in methylation levels within the promoter region observed between OSE and the ovarian tumor samples was not correlated with differences in *HLA-G *expression.

**Table 3:** Real-time RT-PCR analysis of *HLA-G *expression in ovarian samples.

ADENOCARCINOMAS			ADENOMAS		NORMAL	
Patient	Expression	FIGO Stage	Patient	Expression	Patient	Expression
242	0.42	IIIc	386	0.67	500	3.50
369	3.05	IIIc	371	0.83	492	20.53
183	4.03	IIIc	416	3.54	525	53.71
212	177.01	IIIc	382	13.36		

AVE	46.13		AVE	4.60	AVE	25.91
SD	87.27		SD	5.98	SD	25.53

## Discussion

Contrary to other HLA molecules, *HLA-G *regulation is only partially understood. Studies of the methylation levels of 450 bp of the promoter region of *HLA-G *in melanoma and choriocarcinoma cell lines showed a correlation between expression and low levels of methylation [15]. In our study we looked at the same region in the ovarian cancer cell line BG-1, which showed almost complete methylation of all CpG dinucleotides and no expression of *HLA-G*. Treatment of the cells with the methylation inhibitor, 5-aza-dC, resulted in a significant decrease in levels of methylation at the *HLA-G *promoter region that correlated with an increase in levels of *HLA-G *expression (Figures 1 and 2). Consistent with previously published studies conducted with other cancer cell lines [14,15], our results suggest that the methylation status of the *HLA-G *promoter region may be important for the control of *HLA-G *expression in ovarian cancer.

To test this hypothesis in tissues, we analyzed methylation of the *HLA-G *5' regulatory region in 4 OSE samples and 18 malignant and benign ovarian tumor samples. In addition, we measured the relative levels of *HLA-G *expression in 11 OSE and ovarian tumor samples from which cancer cells were isolated using laser capture microdissection (LCM). We found that there were significant differences in levels of methylation within the 5' *HLA-G *regulatory region between OSE and the malignant and benign ovarian tumor samples, but no significant difference between the malignant and benign tumor samples. This observation is consistent with the view that any putative adaptive advantage imparted to tumor cells by *HLA-G *apparently does not distinguish between malignant and benign tumor cells.

We found that many of the CpG sites displaying a significant decrease in methylation in the tumor samples were located in proximity to intact binding sites of regulatory proteins known to serve as activators of gene expression (CCAAT box -76 and an intact SP1 and RFX binding site within the X1 box at -130 [13,19]; Figure 3). Hypomethylation of these sites in ovarian tumors may serve to potentiate *HLA-G *expression. In contrast, we observed a significant increase in levels of methylation in tumor samples at CpG sites located in proximity to a hypoxia response element [16] located 243 bp upstream of the *HLA-G *transcriptional start site. Interestingly, this same region remained methylated in 5-aza-dC treated BG-1 cells, suggesting that strong selection may operate to prevent binding to the HRE in ovarian tumor cells.

HRE is the binding site of HIF-1, a protein that plays a critical role in the cellular response to hypoxia, with the potential to act as either a positive or negative regulatory factor [20-22]. Evidence has recently been reported that HIF-1 may act as a negative regulator of *HLA-G *expression in some human melanoma (FON) and choriocarcinoma (JEG-3) cell lines [22] The functional significance of the methylation of the CpG island surrounding the HIF-1 binding site in the ovarian tumor samples remains to be determined. However, preventing the binding of a potential repressor protein (HIF-1) may help maintain the transcription of *HLA-G *in ovarian tumors under hypoxic conditions thus allowing the tumor cells to evade cytotoxic T lymphocyte recognition and destruction.

It is important to keep in mind that the postulated significance of methylation changes in gene regulatory regions is the increased (hypomethylation) or decreased (hypermethylation) access it provides for the binding of proteins or protein complexes that regulate gene expression [23]. Thus, although methylation changes in gene regulatory regions may be necessary for subsequent changes in levels of expression, these changes in methylation alone may not be sufficient to effect expression changes. The fact that the significant differences in methylation we observed between OSE and ovarian tumor samples do not correlate with differences in *HLA-G *expression may serve to underlie this distinction. Our results are consistent with a scenario whereby changes in methylation in ovarian tumor cells potentiate them for *HLA-G *transcriptional activation (or avoidance of transcriptional repression) by regulatory proteins induced by micro-environmental or mutational changes in specific tumor cell lineages.

## Conclusion

Differences in methylation within the 5' regulatory region of *HLA-G *were detected between normal ovarian surface epithelial cells and ovarian cancers. The most striking difference was in a region (-211 to -290 from *HLA-G *TSS), containing an intact hypoxia response element (HRE). This region was completely methylated in all of the ovarian tumor (malignant and benign) samples examined, but was only variably methylated in normal surface epithelial cells. Interestingly, this same region remained completely methylated in an ovarian cancer cell line (BG-1) after treatment with 5-aza-dC suggesting that there is strong selection against loss of methylation in this region in these ovarian cancer cells. Since HRE is the binding site of a known repressor of *HLA-G *expression (HIF-1), we hypothesize that methylation of the region surrounding the HRE may help maintain the potential for expression of *HLA-G *in ovarian tumors. The fact that no correlation exists between methylation and *HLA-G *gene expression between ovarian tumor samples and OSE, leads us to conclude that changes in methylation may be necessary but not sufficient for *HLA-G *expression in ovarian cancer.

## Methods

### Tissue samples

Ovarian tumor samples were collected at Northside Hospital (Atlanta, GA) during surgery and snap frozen in liquid nitrogen within 1 minute of removal from patients. Brushings of normal ovarian surface epithelial cells (OSE) were preserved in RNAlater (Ambion, Austin, GA) within 1 minute of removal from patients. Patient consent and approval from the Institutional Review Boards of the University of Georgia, Georgia Institute of Technology, and Northside Hospital were obtained. Pathological and clinical information of samples is presented in Table 4.

**Table 4:** Patient samples analyzed in this study

Patient	Histology	Age	FIGO Stage	Grade
228	Clear cell adenocarcinoma	55	Ia	N/A
317	Serous papillary adenocarcinoma	59	Ic	3
170	Mixed endometrioid/serous papillary adenocarcinoma	57	IIa	2
413	Serous papillary adenocarcinoma	50	IIb	3
212	Mixed endometrioid/serous papillary adenocarcinoma	59	IIIc	3
369	Serous papillary adenocarcinoma	52	IIIc	3
242	Serous papillary adenocarcinoma	64	IIIc	3
229	Serous papillary adenocarcinoma	58	IIIc	3
183	Serous papillary adenocarcinoma	66	IIIc	2
412	Mucinous cystadenoma	65		
377	Mucinous cystadenoma	42		
383	Mucinous cystadenoma with focal borderline features	75		
382	Serous cystadenofibroma	67		
386	Serous cystadenofibroma	59		
388	Serous cystadenofibroma	53		
371	Serous cystadenofibroma	60		
400	Serous cystadenofibroma	43		
416	Serous cystadenoma	65		
492	Normal surface epithelium	38		
499	Normal surface epithelium	69		
500	Normal surface epithelium	25		
525	Normal surface epithelium	70		

### Human cell-line culture

The BG-1 ovarian adenocarcinoma cell line was kindly provided by JM Hall and KS Korach (NIH). Cells were propagated in DMEM: F12/50:50 (Invitrogen, Carlsbad, CA), supplemented with 2 mM L-glutamine (Mediatech Inc., Herndon, VA), 10% heat inactivated FBS (Invitrogen, Carlsbad, CA), 1 mM sodium pyruvate (Mediatech Inc., Herndon, VA), and 1% penicillin and streptomycin (Mediatech, Inc., Herndon, VA) at 37°C, 5% CO^2 ^and ~80% relative humidity.

### Cell treatments

BG-1 cells were plated at low density 16 hours before treatment with 50 μM 5-aza-deoxycytidine (Sigma, St. Louis, MO) for 5 days. On the third day of treatment, the media was replaced with fresh media containing 5-aza-dC. Control cells treated with PBS were grown for the same amount of time.

### Laser capture microdissection (LCM)

Fresh frozen tissues from tumors were cut into seven-micron sections, applied to non-charged slides, then fixed in 75% ethanol for 30 seconds, stained and dehydrated using the HistoGene LCM Frozen Section Staining Kit (Arcturus, Mountain View, CA). LCM was performed with an AutoPix Automated Laser Capture Microdissection System using the CapSure Macro Caps (Arcturus, Mountain View, CA). Approximately 10,000 cells were captured on each of 3–4 caps per sample. Subsequently the DNA was extracted from captured cells using the PicoPure DNA Extraction Kit (Arcturus, Mountain View, CA). RNA extraction from captured cells was carried out using the PicoPure RNA Extraction Kit and amplified with the RiboAmpHS RNA Amplification Kit (Arcturus, Mountain View, CA).

### DNA and RNA extraction from ovarian surface epithelial cells

Epithelial brushings were obtained from normal ovaries and kept in RNAlater (Qiagen, Valencia, CA). For DNA extraction, cells were spun down and re-suspended in lysis buffer (10 mM Tris HCl, 10 mM EDTA, 0.2% SDS and 50 mM NaCl), with 50 μg/ml RNAse A (Invitrogen, Carlsbad, CA) and 100 μg/ml Proteinase K (EM Science, Gibbstown, NJ) overnight at 37°C. DNA was then phenol-extracted and ethanol precipitated. For RNA extraction, cells were spun down and Trizol was added, following the manufacturer's recommended protocol (Invitrogen, Carlsbad, CA). cDNA was synthesized from RNA using the RiboAmpHS RNA Amplification Kit (Arcturus, Mountain View, CA).

### RT-PCR

#### Semi-quantitative

Total RNA was extracted from BG-1 cells by adding 1 ml of Trizol (Invitrogen, Carlsbad, CA) according to the manufacturer's recommendations, and further purified using RNEasy Mini Kit (Qiagen, Valencia, CA). Four to six μg of DNAse-treated RNA (DNA-free; Ambion, Houston, TX) were used to generate cDNA by oligo-dT reverse transcription with the Superscript RT-PCR Kit (Invitrogen, Carlsbad, CA). Primers that recognize all *HLA-G *isoforms were designed from the *HLA-G *RNA reference sequence (X12273) at 257F 5'-GGAAGAGGAGACACGGAACA-3' and 1200R 5'-TCCTGTTCCAGAAAAGGGG-3'. *GAPDH *(NM_002046) was used as an endogenous control, using primers 287F 5'-GAAATCCCATCACCATCTTCCAG-3' and 599R 5'-ATGAGTCCTTCCACGATACAAAAG-3'.

#### Real-time RT-PCR

Total RNA extracted from LCM captured ovarian tumor cells and normal OSE cell was converted to amplified cDNA for real-time RT-PCR. TaqMan Gene Expression Assays (Applied Biosystems, Foster City, CA) were conducted following manufacturer's protocol for *HLA-G *(Hs00365950_g1) and for *GAPDH *endogenous control (Hs99999905_m1) using the DNA Engine Opticon System (MJ Research, Waltham, MA). A standard curve of serial dilutions of a plasmid containing either *HLA-G *or *GAPDH *was established for each assay to obtain a relative quantification of *HLA-G *or *GAPDH *gene expression. For each sample normalized *HLA-G *expression values are presented as the ratio between the target gene (*HLA-G*) and the endogenous control *(GAPDH*).

### Sodium bisulfite genomic sequencing

Five hundred nanograms to one microgram of genomic DNA from the BG-1 cells, LCM captured ovarian tumor cells and normal OSE cells were modified with sodium bisulfite according to Herman et al. [24]. For LCM samples, sodium bisulfite modification was performed by pooling DNA from 2 to 3 caps per patient sample, and adding 15 μl of hydroquinone and 200 μl of sodium bisulfite, as well as 1 μg of Herring Sperm DNA (Promega, Madison, WI) as a carrier.

Modified DNA was used for PCR with primers for the CpG-enriched 5' regulatory region of *HLA-G *[15]. Position of the primers from the ATG codon of the *HLA-G *gene are: Primer F (-456) 5'-AAGAGTATAGGAGGATAGGTAAGG-3' and primer R (-16) 5'-AACACCATAACCACCATCCTTAAC-3'.

A second region of *HLA-G *from -7 to +392 from the ATG codon was amplified performing two rounds of PCR. The first round was done with primers F-5'-GGATTTATTTTTTTTAGATGTTAAG-3' and R-5'-ATCTACAAATTCATTCTATCAATCTATAC-3', and then nested PCR was performed with F-5'-GTTAAGGATGGTGGTTATGGTGTT-3' and R-5'-TATCTCCTCTTCCCAATACTCCAA-3'. PCR products were purified using the QIAquick Gel Extraction Kit (Qiagen, Valencia, CA), and the DNA cloned using TOPO TA Cloning for Sequencing Kit (Invitrogen, Carlsbad, CA). DNA plasmids from individual colonies were extracted using QIAprep Spin Miniprep Kit (Qiagen, Valencia, CA). Automated sequencing was performed by Integrated Biotech Laboratories (Athens, GA).

## Competing interests

The authors declare that they have no competing interests.

## Authors' contributions

LM, JM Conceived and designed the experiments, LM, LDW, LVM Performed the experiments, LM, JFM, LDW Analyzed the data, KAT, BB Contributed reagents/materials,  LM, JFM, LDW Drafted the manuscript:. All authors read and approved the final manuscript.

## References

[B1] Kovats S, Main EK, Librach C, Stubblebine M, Fisher SJ, DeMars R (1990). A class I antigen, HLA-G, expressed in human trophoblasts. Science.

[B2] Carosella ED, Moreau P, Le Maoult J, Le Discorde M, Dausset J, Rouas-Freiss N (2003). HLA-G molecules: from maternal-fetal tolerance to tissue acceptance. Adv Immunol.

[B3] Lin A, Yan W-H, Xu H-H, Gan M-F, Cai J-F, Zhu M, Zhou M-Y (2007). HLA-G expression in human ovarian carcinoma counteracts NK cell function. Ann Oncol.

[B4] Lefebvre S, Antoine M, Uzan S, McMaster M, Dausset J, Carosella ED, Paul P (2002). Specific activation of the non-classical class I histocompatibility HLA-G antigen and expression of the ILT2 inhibitory receptor in human breast cancer. J Pathol.

[B5] Bukur J, Malenica B, Huber C, Seliger B (2003). Altered expression of nonclassical HLA class Ib antigens in human renal cell carcinoma and its association with impaired immune response. Hum Immunol.

[B6] Rouas-Freiss N, Moreau P, Ferrone S, Carosella D (2005). HLA-G proteins in cancer: do they provide tumor cells with an escape mechanism?. Cancer Res.

[B7] Sheu JJ-C, Shih IM (2007). Clinical and biological significance of HLA-G expression in ovarian cancer. Semin Cancer Biol.

[B8] Paul P, Rouas-Freiss N, Khalil-Daher I, Moreau P, Riteau B, Le Gal FA, Avril MF, Dausset J, Guillet JG, Carosella ED (1998). HLA-G expression in melanoma: a way for tumor cells to escape from immunosurveillance. Proc Natl Acad Sci USA.

[B9] Rouas-Freiss N, Moreau P, Menier C, LeMaoult J, Carosella D (2007). Expression of tolerogenic HLA-G molecules in cancer prevents antitumor responses. Semin Cancer Biol.

[B10] Real LM, Cabrera T, Collado A, Jimenez P, Garcia A, Ruiz-Cabello F, Garrido F (1999). Expression of HLA G in human tumors is not a frequent event. Int J Cancer.

[B11] Gobin SJ, Keijsers V, Cheong C, van Zutphen M, Elsen PJ Van den (1999). Transcriptional regulation of HLA-G. Transplant Proc.

[B12] Solier C, Mallet V, Lenfant F, Bertrand A, Huchenq A, Le Bouteiller P (2001). HLA-G unique promoter region: functional implications. Immunogenetics.

[B13] Tripathi P, Agrawal S (2006). Non-classical HLA-G antigen and its role in the cancer progression. Cancer Invest.

[B14] Moreau P, Mouillot G, Rousseau P, Marcou C, Dausset J, Carosella ED (2003). HLA-G gene repression is reversed by demethylation. Proc Natl Acad Sci USA.

[B15] Mouillot G, Marcou C, Rousseau P, Rouas-Freiss N, Carosella ED, Moreau P (2005). HLA-G gene activation in tumor cells involves cis-acting epigenetic changes. Int J Cancer.

[B16] Chang C-C, Ferrone S (2003). HLA-G in melanoma: can the current controversies be solved?. Semin Cancer Biol.

[B17] Auersperg N, Wong AST, Choi K-C, Kang SK, Leung PCK (2001). Ovarian surface epithelium: Biology, endocrinology, and pathology. Endocr Rev.

[B18] Shih I-M, Kurman RJ (2004). Ovarian tumorigenesis: a proposed model based on morphological and molecular genetic analysis. Amer J Path.

[B19] Rousseau P, Paul P, O'Brien M, Dausset J, Carosella ED, Moreau P (2000). The X1 box of HLA-G promoter is a target for RFX and Sp 1 factors. Human Immunol.

[B20] Manalo DJ, rowan A, Lavoie T, Natarajan L, Kelly BD, Ye SQ, Garcia JGN, Semenza GL (2005). Transcriptional regulation of vascular endothelial cell responses to hypoxia by HIF-1. Blood.

[B21] Eltzchig HK, Abdulla P, Hoffman E, Hamilton KE, Daniels D (2005). HIF-1-dependent repression of equilibrative nucleoside transporter (ENT) in hypoxia. J Exp Med.

[B22] Mouillot G, Marcou C, Zidi I, Guillard C, Sangrouber D, Carosella ED, Moreau P (2007). Hypoxia modulates HLA-G gene expression in tumor cells. Hum Immunol.

[B23] D'Alessio AC, Szyf M (2006). Epigenetic têtê-à-têtê: the bilateral relationship between chromatin modifications and DNA methylation. Biochem Cell Biol.

[B24] Herman JG, Graff JR, Myohanen S, Nelkin BD, Baylin SB (1996). Methylation-specific PCR: a novel PCR assay for methylation status of CpG islands. Proc Natl Acad Sci USA.

